# Beyond CAG Repeats: The Multifaceted Role of Genetics in Huntington Disease

**DOI:** 10.3390/genes15060807

**Published:** 2024-06-19

**Authors:** Marta Pengo, Ferdinando Squitieri

**Affiliations:** 1 Department of Molecular and Translational Medicine, University of Brescia, 25121 Brescia, Italy; marta.pengo4@gmail.com; 2 Centre for Neurological Rare Diseases (CMNR), Fondazione Lega Italiana Ricerca Huntington (LIRH), 00161 Rome, Italy; 3 Huntington and Rare Diseases Unit, IRCCS Casa Sollievo della Sofferenza, 71013 San Giovanni Rotondo, Italy

**Keywords:** gene modifiers, DNA mismatch repair, loss of interruption, somatic mutations, somatic instability, RNA toxicity, mtDNA, epigenetics

## Abstract

Huntington disease (HD) is a dominantly inherited neurodegenerative disorder caused by a CAG expansion on the huntingtin (*HTT*) gene and is characterized by progressive motor, cognitive, and neuropsychiatric decline. Recently, new genetic factors besides CAG repeats have been implicated in the disease pathogenesis. Most genetic modifiers are involved in DNA repair pathways and, as the cause of the loss of CAA interruption in the *HTT* gene, they exert their main influence through somatic expansion. However, this mechanism might not be the only driver of HD pathogenesis, and future studies are warranted in this field. The aim of the present review is to dissect the many faces of genetics in HD pathogenesis, from cis- and trans-acting genetic modifiers to RNA toxicity, mitochondrial DNA mutations, and epigenetics factors. Exploring genetic modifiers of HD onset and progression appears crucial to elucidate not only disease pathogenesis, but also to improve disease prediction and prevention, develop biomarkers of disease progression and response to therapies, and recognize new therapeutic opportunities. Since the same genetic mechanisms are also described in other repeat expansion diseases, their implications might encompass the whole spectrum of these disorders.

## 1. Introduction

Huntington disease (HD) is a dominantly inherited neurodegenerative disorder characterized by motor, cognitive, and neuropsychiatric features [1]. It is caused by an expanded CAG repeat in exon 1 of the huntingtin (*HTT*) gene [2]. The CAG repeat length explains around 50–70% of the variability in the age at onset (AAO) of HD [3,4]. In fact, two individuals with an identical CAG repeat length can develop HD symptoms decades apart [5]. This is particularly evident in individuals carrying reduced penetrance alleles ranging from 36 to 39 CAG, who might develop HD at very late ages [6].

The unexplained variability in HD onset and pathogenesis underscores the presence of additional genetic factors that exert a crucial influence [7,8,9]. This highlights the necessity for further investigation into the intricate genetic landscape contributing to HD.

In the last few years, dramatic advances in sequencing technology and the availability of larger patient cohorts have moved the HD genetic field forward, identifying modifiers on chromosomes 8 and 15 mainly involved in the DNA mismatch repair pathway [10,11,12]. In addition, cis-acting factors in the *HTT* locus have also been discovered [13] which can modulate disease frequency [14,15] and expressivity [16,17]: particularly, the loss of the CAA interruption variant has raised great interest, especially in carriers of reduced penetrance alleles [18,19,20,21,22].

Genetic modifiers exert their main effect through somatic expansions of the CAG triplet, which appears to be a key driver of HD pathogenesis. Indeed, the *HTT* CAG displays somatic instability, which is greater in the brain and in areas most affected by HD (i.e., particularly the caudate) [8,23,24,25,26]. Research of surrogates of somatic expansion in peripheral tissues is mandatory, as it has been demonstrated that higher blood DNA CAG somatic expansions are associated with worse outcomes [27]. RNA-related pathology, mitochondrial DNA mutations, and epigenetic alterations are emerging aspects in HD genetics research that might contribute to the disentangling of the disease pathogenesis, as well as the identification of potential biomarkers of early disease stages and disease progression, and therapeutic targets [28,29].

In this review, we first provide an overview of the complex role of genetics in HD, in addition to the expanded CAG repeat mutation. We describe gene modifiers including cis- and trans-acting candidate genetic alterations that are thought to have a relevant role in HD AAO and progression.

As the ultimate objective to further improve knowledge in this field, we focus on targets for therapeutic interventions. The contribution of genetics to the development of therapies is fundamental if we consider that drug trials supported by genetics can potentially double clinical success rates [30,31] and two-thirds of 2021 FDA-approved drugs are supported by human genetics evidence [32].

Finally, the implications of pharmaceutical research into genetic modifiers of HD can be extrapolated to encompass other CAG/polyglutamine expansion disorders that share genetic similarities with HD, as will also be elucidated in this review.

## 2. *Cis*-Acting Genetic Modifiers

The typical sequence of the *HTT* repeat tract accounts for a number of CAG repeats, followed downstream by a 12-base-pair interrupting sequence (CAA-CAG-CCG-CCA) and a subsequent CCG tract.

According to the traditional view of the disease, HD pathogenesis has always been related to the toxic effect of the protein through the Poly-Q tract in the huntingtin protein. Since both CAG and CAA encode glutamine, a change in the final CAG-CAA sequence in the *HTT* locus was assumed to be irrelevant. Notwithstanding this, three concurrent genetic studies supported that CAG length is more predictive than polyglutamine length for HD AAO [12,18,19]. The Genome Wide Association Study (GWAS) on genetic modifiers from the GeM-HD consortium detected two association signals at the *HTT* locus influencing AAO [12]. The other two studies identified a variant characterized by the loss of the CAA-CAG sequence at the end of the *HTT* locus, thus extending the uninterrupted CAG length, leaving the polyglutamine length unchanged [12,18,19]. This variant was defined as the loss of interruption (LOI) and was associated with a hastened age of onset of approximately 9 years [19] Conversely, a duplication of the interruption was associated with the opposite effect, delaying the AAO.

Somatic repeat instability is the most probable mechanism mediating the LOI effect in low penetrance alleles since they were associated with increased somatic expansions in the blood and in the germline [19]. However, somatic instability might not be the only driver of the LOI. Indeed, possible alternative pathogenic processes have been suggested, such as repeat-associated non-AUG-dependent translation, CAG RNA toxicity (which will be described later in the text), and spliceosome dysregulation [33,34,35,36].

LOI has implications at different levels. From a clinical point of view, although rare (0.9% to 2.5% in fully penetrant alleles [19], LOI is especially important to certain patient sub-populations, in particular in reduced penetrance allele carriers (i.e., CAG 36-39) [37,38]. Approximately a third of symptomatic carriers of low penetrance alleles harbor this variant [19,20]. As a consequence, LOI influence could be taken into account by genetic counseling guidelines in the future when patients carry low penetrance alleles [39]. At a diagnostic level, it should be considered that current diagnostic tests do not recognize them, since they only assess the length of amplified products and do not take DNA sequence into account [40]. Thus, developing an LOI-specific PCR assay may complement genetic testing in the future. Finally, interruptions represent a potential therapeutic target, not only in HD but also for other repeat expansion disorders [41,42,43]. At present, different therapeutic approaches are under investigation to interfere with somatic repeat instability in preclinical studies; the CRISPR-Cas9 technique might be used to introduce additional interruptions into the CAG tract [44,45].

Future aims in this research field might be the investigation of LOI in different population groups other than Caucasians, such as individuals from Africa and Asia. The importance of studying genetically diverse populations in the context of the disease has been recently highlighted in people of African ancestry [15,17]. More in detail, atypical allele structures characterized by either a CCG-CCA proline loss downstream to the CAG repeat [17] or by a rare haplotype recombination [15] were observed in African disease alleles, whereas they were rare or missing in people with European ancestries. In these cases an association with an anticipation of an expected AAO of approximately 7.1 years in both African and European populations [17] and an increased frequency of juvenile cases in Middle East populations of African ancestries were observed [15,46]. Moreover, a linkage disequilibrium between the number of CAA and the number of CCA variants was reported, and a double loss was associated with a much earlier AAO. CCG-CCA loss seemed to act through a mechanism different from somatic instability since it was associated with reduced somatic expansion in blood DNA [17].

These recent findings demonstrate that not only the number of CAG repeats and the polyglutamine are involved in HD pathogenesis, but there might be also other factors, such as the downstream polyproline sequence which might affect the mRNA and the encoded protein. These alternative alleles warrant further investigation since they might influence the AAO, and analyzing them in parallel with CAG length will have a great impact on genetic counseling [17].

## 3. *Trans*-Acting Genetic Modifiers: DNA Mismatch Repair Pathway Genes

**Figure 1:**
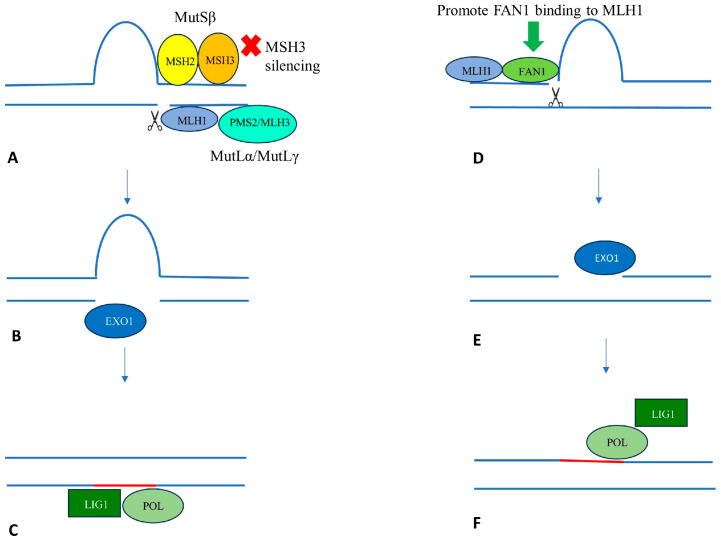
The role of DNA mismatch repair in repeat expansion and possible therapeutic targets. Left (**A**–**C**): MSH3 mediated repair at a CAG loopout, leading to repeat expansion. Right (**D**–**F**): FAN1 mediated repair preventing repeat expansion. FAN1 protects against repeat expansion through a mechanism that depends on its nuclease activity and binding to MLH1. See text for details.

Several GWAS conducted in the last years on large international cohorts allowed the identification of other genetic factors influencing HD AAO besides CAG length (Table 1, Figure 1). Many of them fall under the umbrella of DNA repair genes, and more specifically are involved in the mismatch repair pathway. The Genetic Modifiers of Huntington’s Disease (GeM-HD) consortium’s GWAS conducted on 4082 HD patients identified three significant modifying signals at two loci, one on chromosome 8 and the others on chromosome 15, associated with the AAO [10].

Two independent signals on chromosome 15 corresponded to the gene encoding *FAN1*, which is an endo- and exonuclease involved in interstrand DNA crosslink repair [47]. *FAN1* seems to be protective in HD: *FAN1* depletion in the neurons of animal models and in HD patients accelerates repeat expansion [48]. In fact, *FAN1* variants that cause protein decreases hastened the disease onset by more than 6 years earlier than would be expected from CAG length alone, and the others that increase *FAN1* expression delayed the disease onset by 1.4 years [48]. The current thinking is that *FAN1* suppresses somatic expansion at the *HTT* locus [49,50]. Two mechanisms have been shown to mediate somatic stability in *HTT*. The first is mediated by MLH1 binding [48,50,51]; indeed, FAN1 binds to MLH1, preventing its recruitment in the mismatch repair complex, thus stabilizing CAG repeat expansion [51]. The interaction between FAN1 and MLH1 is negatively regulated by phosphorylation of FAN1’s S126 residue [48]. The second is related to FAN1’s nuclease activity [52,53] which is activated by PCNA and RFC on DNA harboring triplet repeat extrusions [54]. McAllister and colleagues described *FAN1* mutations to be associated with a worse HD onset and a more severe phenotype. In particular, AAO-hastening SNPs (Single Nucleotide Polymorphisms) demonstrated reduced nuclease activity [53].

*FAN1* is also involved in other neurogenerative diseases caused by repeat expansion, such as certain hereditary spinocerebellar ataxias and X fragile syndrome [55,56,57]. Therefore, the DNA repair pathway represents a common genetic mechanism underlying repeat expansion disorders with potential broader therapeutic implications [58]. Moreover, there is also evidence of the involvement of *FAN1* in epilepsy, bipolar disorder, schizophrenia, and autism [58]. Future works are needed to assess the therapeutic tractability of *FAN1* in attenuating *HTT* somatic expansion. A possible therapeutic target under current investigation might be the aforementioned phosphorylation of FAN1-S126.

The chromosome 8 signal observed in GeM-HD GWAS was associated with an anticipated AAO of 1.6 years earlier than expected and could correspond to *RRM2B* or *UBR5* loci. These genes are involved in the following functions implicated in HD pathogenesis: mitochondrial regulation, DNA maintenance, oxidative stress, and proteostasis.

Another study [59] confirmed the signals identified on chromosomes 8 and 15 and also found a locus at *MLH1* on chromosome 3, associated with a delay in disease onset of 0.7 years. Dominant loss of function mutations of *MLH1* are associated with Lynch syndrome [60], the most common cause of hereditary colorectal cancer; moreover, the *MLH1* gene is associated with brain CAG instability in the *Htt* knock-in mouse [61].

The GWAS study conducted on 216 and 1773 participants from the TRACK-HD and REGISTRY studies, respectively, identified an association at chromosome 5, which corresponds to *MSH3*, associated with slower disease progression [11]. As with *FAN1* and *MLH1*, *MSH3* is also involved in the DNA mismatch repair pathway [62,63,64] and is the first identified genetic modifier of the rate of progression in HD. In particular, Moss and colleagues demonstrated that each copy of the minor allele at the lead SNP in *MHS3* was associated with a reduction in the change in the Unified Huntington’s Disease Rating Scale (UHDRS) Total Motor Score and Total Functional Capacity (0.4 and 0.12 units per year, respectively). *MSH3* involvement has been demonstrated also in myotonic dystrophy type 1 [65], further underlying the common genetic background of triplet diseases. O’Reilly and collaborators have recently characterized a fully chemically modified short interfering RNA (siRNA) capable of silencing *MSH3* in both in vitro and in vivo models [66]. The application of siRNA to downregulate *MSH3* proved highly effective in inhibiting the expansion of CAG repeats within the striatum in two distinct HD murine models. This discovery presents a promising therapeutic avenue for individuals afflicted by HD and other disorders characterized by repeat expansion mutations.

Interestingly, Lee and colleagues suggested that genetic modifiers might preferentially affect motor or cognitive functions [67]. For instance, *MSH3* could mainly impact the cognitive domain, whereas *FAN1* could impact motor function. As a consequence, these genetic modifiers might act differentially on the neuronal networks underlying diverse clinical outcomes.

In 2019, an extended GWAS study involving over 9000 HD patients from the REGISTRY and Enroll-HD cohorts confirmed the findings of the previous GeM-HD GWAS and also identified new HD onset-associated loci, corresponding to other DNA repair genes *PMS1*, *MSH3*/*DHFR*, *PMS2,* and *LIG1*, as well as *TCERG1* and *CCDC82* [12]. Interestingly, *PMS1* has been found to be a target of splice modulators, small molecules that are being investigated in HD clinical trials to reduce HTT levels, providing alternative targets to prevent CAG somatic expansion [68].

The results of these genetic studies point out the central role of the mismatch DNA repair pathway in HD pathogenesis (Figure 1). Expanded repeat sequences can form secondary structures, such as hairpins and large loops, that might induce the mismatch repair complex to act erroneously, leading to somatic expansions. In more detail, MutSβ (MSH2-MSH3) recognizes these structures and recruits MutLα (MLH1-PMS2) or MutLγ (MLH1-MLH3), endonucleases that co-ordinate excision [69]. The MutL complex erroneously creates a break in the strand opposite the loop. The polymerase then uses the strand with the loop as the template strand, thus determining the elongation of the repeat sequence. On the contrary, MutSα (MHS2-MSH3) seems not to be involved in CAG instability, since it recognizes small DNA loops, rather than the longer loops targeted by MutSβ.

MSH2, MSH6, or MLH1 depletion may cause cancer in humans, while MSH3 loss of function does not affect lifespan or cause cancer in mice [70]. Therefore, small molecules or ASOs against MSH3 might represent fruitful strategies for potential therapies for HD [71].

DNA-repair genes represent promising candidates for future therapies, and in preclinical development, MSH3 is the most encouraging target [62,72]. Moreover, since slipped DNAs occur during somatic repeat expansions, small molecules that specifically bind to these structures are under development [36].

**Table 1:** Genetic modifiers implicated in HD onset and progression.

Genes	Effect
FAN1	FAN1 depletion in animal models and humans accelerates repeat expansion [48]. Increased expression promotes CAG repeat stability and is associated with delayed disease onset [49,50,55]. Variants that delay or hasten disease onset [10,53].
MLH1	The MLH1 gene is associated with brain CAG instability in Htt knock-in mice [61]. MLH1 delays disease onset [59].
MSH3	Promotes somatic CAG expansions, thus contributing to an earlier onset [62,63,64]. Variants are implicated in disease progression [11].
PMS1, PMS2, LIG1	Variants alter disease onset [12].

## 4. Somatic Mutations and Mosaicism

Whereas inherited mutations are transmitted across generations, somatic mutations occur post-zygotically. They can develop throughout the entire life of an individual, leading to somatic mosaicism, a condition in which only some cells of an individual harbor the mutation [73,74]. Their role in contributing to disease pathogenesis has been first described in cancer. Recently, thanks to technical improvements such as single-cell and whole-genome sequencing, growing evidence has supported their involvement also in neurodevelopmental disorders [75,76], such as in brain malformations associated with epilepsy and intellectual disabilities [77]. Besides these conditions, it has been demonstrated that somatic mutations are involved also in normal brain aging [78] and neurodegenerative disorders [79].

The CAG repeat causing HD shows great meiotic instability and frequently increases in length across generations [80,81]. The risk of expansion is higher in spermatogenesis than in oogenesis. This may contribute to partially explain pediatric-onset HD cases inherited by affected fathers [81,82], and the occurrence of de novo mutations from paternal intermediate alleles [83].

CAG repeat expansion is highly unstable not only in germline but also in somatic cells, thus determining somatic mosaicism. The tissue specificity of somatic repeat instability has been described in both mice models and in humans, with the striatum and cerebral cortex displaying the highest levels of somatic expansions [84,85,86,87,88,89,90]. Brain somatic CAG instability is associated with an earlier age at onset [90] and mounting evidence suggests that the degree of somatic repeat length in undifferentiated neurons better explains the AAO than the germline repeat [72].

Somatic instability can be investigated in peripheral tissues [27] whose alterations might mirror what happens in the brain. In a large study of nearly 750 HD mutation carriers, somatic expansions in the blood correlated with worse clinical outcomes, encompassing an earlier AAO, worse baseline motor scores, and higher disease progression scores [18]. Variants in several DNA repair genes are associated with somatic expansion, both in HD animal models [61,62,91] and HD patients [12,18,64]. Therefore, it seems reasonable that their effect might be mediated through somatic expansion of the CAG repeat. However, other factors might also contribute to drive somatic instability. A possible candidate is oxidative stress since it could modify instability rates [92]. Somatic expansions in blood samples increase with age, whereas they are minimal in fetal mutation carriers [93]. As a consequence, these findings support a computational, simulational approach proposed by Kaplan et al. to explain the onset and progression of HD [94]. According to this view, disease onset occurs once the repeat sequence has increased in length beyond a cell type-specific pathological threshold in a critical proportion of vulnerable cells to trigger toxicity and dysfunction, leading eventually to cell death [94,95]. The threshold for each specific cell type is yet to be determined. By performing quantitative analyses of CAG instability across several central nervous system (CNS) regions and peripheral post mortem tissues of HD individuals, Pinto et al. demonstrated that *HTT* CAG repeat expansion indeed occurs in all tissues analyzed, though to different extents [25]. The greatest instability was found in multiple cortical regions and neostriatum in the CNS, and liver in the periphery, the latter of which indeed affected children with highly expanded and unstable mutations [96].

The presence of CAG instability also in peripheral cells opens new ways to further investigate triplet mosaicism. Furthermore, given the central role of somatic expansions in disease progression, targeting repeat instability might have extremely relevant, even though challenging, therapeutic implications in triplet disorders [97].

Altogether, the above considerations lend support to the following hypotheses: (1) the inherited expanded *HTT*-CAG repeats undergo further expansion somatically toward a critical threshold length in vulnerable cell types [91], thus extensively and progressively affecting HD biology in a length-dependent manner [98]; (2) when the expanded CAG threshold length is reached, a mechanism is triggered that affects HD progression [94,98]; (3) in cis haplotypes may affect HD frequency and toxicity of mutant HTT in axons [13,14,15]; (4) factors in trans to expanded *HTT*-CAG repeats may act as disease modifiers [9,10,11,12].

## 5. RNA-Related Pathology

Although a great number of works demonstrated that the mHTT protein affects many cellular functions, leading to cell death and neurodegeneration, an increasing body of evidence indicates that also the mHTT RNA may contribute to toxicity [34].

Normally, two alternatively spliced transcripts emanate from the *HTT* gene. These transcripts vary in the length of their 3′ untranslated region (UTR) by 3 kilobases (kb), producing an identical HTT protein. The extended transcript is primarily observed in the brain, whereas the other variant has a broader expression throughout various tissues [99]. However, a well-documented feature of HD is that HTT pre-mRNA splicing can be altered, leading to the production of different isoforms of the huntingtin protein [100,101]. Among the multiple shorter versions of the HTT, exon 1 is the most toxic N-terminal fragment [102,103]. This short exon 1 transcript (HTT1a) was described in mice models and post mortem brains of subjects with HD [104]; its level is proportional to the CAG repeat length, it is only seen in mutant alleles, and it produces the pathogenic and highly aggregation-prone exon 1 huntingtin protein [105]. In mice models expressing the human *HTT* gene (YAC128 mice), HTT1a was found both in large nuclear RNA clusters and as single transcripts in the cytoplasm [106]. The levels of exon 1 HTT in YAS128 mice correlated with HTT aggregation, suggesting the hypothesis that exon 1 HTT initiates the aggregation process. These findings might have profound therapeutic implications, underlying the importance of specifically targeting the exon 1 protein instead of the full-length HTT mRNA.

Another characteristic of RNA pathology in HD is related to the production of toxic RNA species. Indeed, repeat-containing RNAs may agglomerate in the nucleus as foci or undergo aberrant repeat-associated non-AUG (RAN) translation. RAN translation refers to a phenomenon in which RNA sequences containing repetitive elements are translated into proteins without the requirement of a traditional start codon (ATG) that usually initiates protein translation. As a result, short, abnormal proteins with repeated amino acid sequences are produced. This process was documented in certain genetic disorders associated with repeat expansion, such as HD, myotonic dystrophy, and amyotrophic lateral sclerosis [33,107,108]. In HD, these elements accumulate most abundantly in regions associated with HD pathology, such as the striatum, the white matter, and the cerebellum in juvenile HD [108]. RNA translation adds a further layer of complexity to the molecular mechanisms underlying HD and other neurodegenerative diseases. The abnormal proteins generated through RAN translation might contribute to cellular dysfunction and toxicity, ultimately contributing to neurodegeneration [109,110]. However, another study showed that HD knock-in mice lack RAN-mediated toxicity [111]; thus, the role of this form of translation in HD pathogenesis requires further confirmation.

In summary, RNA plays a critical role in the development and progression of HD. The expanded CAG repeat within the *HTT* gene leads to various RNA-related abnormalities, including altered transcription, RNA processing, and the generation of toxic RNA species. Harnessing the understanding of these RNA-related mechanisms has led to the development of potential therapeutic strategies aimed at mitigating the effects of the disease. For instance, pharmaceutical companies have been developing *HTT*-lowering therapies, aiming at specifically targeting and leading to HTT mRNA degradation including small interfering RNA molecules (siRNAs) and antisense oligonucleotides (ASOs). These therapies aim to finally reduce the production of the toxic proteins, potentially slowing down or halting the progression of the disease [1,71].

## 6. Mitochondrial Mutations

Robust evidence demonstrates that mitochondrial dysfunction plays a central role in normal aging and neurodegenerative diseases [112,113]. Mitochondrial DNA (mtDNA), compared to nuclear DNA (nDNA), is 10 times more susceptible to mutations and plays a key role in the development and progression of neurodegenerative disorders, including HD [114]. Additionally, it is worth noting that the repair mechanisms for mtDNA and nDNA differ [115] and there is still a dearth of studies focusing on the assessment of mtDNA repair machinery [116].

Mitochondrial alterations are well documented in HD [117,118] and several molecular mechanisms have been hypothesized to connect mHTT to mitochondrial dysfunction [119]. Disentangling these mechanisms is of particular interest since promoting mitochondrial function might represent a complementary approach to target the causative gene mutation in HD. Mitochondria are crucial for energy metabolism and their proteins are encoded by both nDNA and mtDNA. mtDNA damage has been described in the brain of HD mouse models [120,121], adult HD [122,123], and pediatric HD patients [124]. Moreover, nDNA and mtDNA alterations have been reported also in peripheral tissues, suggesting a possible role as easily accessible biomarkers of disease progression and therapeutic monitoring. In this field, several open issues should be addressed, namely which kind of peripheral cell is the most promising (e.g., different subpopulations of leukocytes), the different roles as potential biomarkers of nDNA and mtDNA, and the correlation with scores of disease severity and disease progression.

Different works have described alterations in mtDNA amounts in leukocytes of HD patients [125,126,127,128]. However, the results of the first studies were not conclusive, showing both a depletion [125,127,128] and an increase in the mtDNA copy number [126,129]. An opposed alteration in mtDNA amount between leukocytes and fibroblasts in the same patients was reported [129]. These discrepancies could have been due to differences in the size of populations, techniques, or type of cells considered. Moreover, an association between mtDNA alterations and disease severity was not clearly defined. On the other hand, results from another study conducted on peripheral blood mononuclear cells (PBMC) of 36 HD patients were further in contrast to the works aforementioned [130]. Askeland and colleagues found a reduction in genes associated with aerobic metabolism in PBMC of HD patients, thus suggesting mitochondrial dysfunction. However, they showed reduced mtDNA damage in HD patients compared to healthy controls, whereas nDNA was severely damaged in patients. An inverse correlation between nDNA damage and total functional capacity (TFC) was described.

A study conducted on a large population of 1549 HD patients shed more light on this research field [28]. A significant increase in mtDNA heteroplasmies of predicted pathogenicity was found in the lymphoblast of HD patients, which correlated with HD stage and disease severity, determined by motor and cognitive scores and TFC. Moreover, with a 6-year longitudinal follow-up of a subgroup of 169 HD patients, the authors found that the expansion of pathogenic mtDNA heteroplasmies was correlated with disease progression, evaluated by means of decline in TFC, motor score, and symbol digit modality test results.

In conclusion, mtDNA alterations in peripheral tissues could provide an accessible biomarker of disease progression in HD. The implications might be even broader in the field of triplet disorders since the observations have been extended also to other polyglutamine diseases [127].

## 7. Epigenetics

Epigenetics describes heritable modifications that alter the accessibility of DNA and regulate gene transcription without changing the underlying DNA sequence. Methylation is the most studied epigenetic alteration and consists of the binding of a methyl group to CpG dinucleotide in the promoter region of a gene, thus reducing its transcription.

Various DNA methylation alterations in neurological diseases are associated with disease activity, disease progression, and clinical outcomes, and may have a prognostic or diagnostic value [131]. Different works underlined the potential role of methylation in HD, both in animal models and in humans. Through the analysis of the cortex DNA methylation profiles in HD patients, De Souza and colleagues found an association between DNA methylation and the age of disease onset [132]. However, they failed to identify any HD-associated DNA methylation changes at probe sites.

Transcriptional dysregulation is a major characteristic of early HD, preceding neuronal death, demonstrated both in human post mortem tissues and mouse models [23,133]. The polyglutamine-expanded *HTT* might be directly involved in these expression changes. Indeed, in cell lines derived from mouse striatal neurons, the presence of mutant HTT was associated with significant changes in DNA methylation, with the downregulation of the expression of genes associated with neurogenesis, and neuronal differentiation, such as SOX2, PAX6, and NES [134]. mHTT might be involved in the regulation of DNA methyltransferases [134,135] and it could also bind directly to DNA, thus driving the recruitment of epigenetic modifiers [136]. In primary neuronal models of HD, mHTT increased the levels of DNA methylation in the promoters of BDNF, an essential neurotrophic factor [137]. Conversely, pharmacological inhibition of DNA methyltransferase decreased methylation, thus restoring BDNF transcription.

If on one hand there is evidence that polyglutamine-expanded HTT is associated with transcription regulation of key genes, on the other hand, the epigenetic status in and around the CAG repeat also plays a relevant role. To the best of our knowledge, at present, the largest study on DNA methylation in HD was conducted by Lu and colleagues in seven DNA sources from three species, namely human, mouse, and sheep models [29]. First of all, they demonstrated that manifest HD, but not premanifest, was associated with increased epigenetic age in human blood DNA. This is in line with previous evidence from human brain samples [138]. Secondly, epigenome-wide association studies found that HTT is the main locus involved in all three species. This finding might help to explore disease pathogenesis. Moreover, investigating whether methylation at this locus contributes to drive somatic expansion would be of great interest in research. Finally, methylation levels at three loci (PEX14, GRIK4, and COX4I2) were significantly associated with motor progression in manifest HD.

Post-translational histone modifications (e.g., acetylation) are also epigenetic events that have been well studied in HD. For instance, a reduction in histone acetylation and in specific loci has been documented in several HD models and human HD biosamples [24]. In addition, there is also evidence that many RNA species can contribute to HD pathogenesis which has been described elsewhere [139,140]. One of these species, i.e., lncRNAs, RNA molecules exceeding 200 nucleotides in length, have been observed to exhibit notably higher expression in the brain, playing intricate roles in a multitude of cellular processes, including functions pertaining to transcriptional regulation and chromatin modulation.

In conclusion, assessing the diagnostic relevance of epigenetic regulations in HD through DNA methylation and RNA species might represent an attractive target for future therapeutic intervention.

## 8. Conclusions and Future Perspectives

The field of genetic modification research has expanded enormously in the last years, significantly enhancing our comprehension of somatic instability as the pivotal driver of HD pathogenesis. This newfound knowledge not only holds profound implications for advancing our understanding of other repeat expansion disorders but also offers a wide range of therapeutic prospects for these diseases. In the near future, it is imperative to delve into alternative pathways of pathogenesis that extend beyond somatic instability and to explore the involvement of other genetic factors such as the ones discussed in this review. These avenues of investigation not only promise to disentangle HD pathogenesis but also present opportunities for the development of disease progression biomarkers and innovative therapeutic targets. The multifaceted nature of HD genetics demands a comprehensive approach, and continued extensive research on genetic modifiers holds the key to unlocking new horizons in our quest to cure this devastating disorder.

## Data Availability

No new data were created or analyzed in this study. Data sharing is not applicable to this article.
